# Evaluation of the Immunomodulatory Effects of a Probiotics and Natural Extract-Based Formulation in Bacterial-Induced Prostatitis

**DOI:** 10.3390/life13020389

**Published:** 2023-01-31

**Authors:** Stefania Murzilli, Vincenzo Mirone, Marta Micheletto, Erik Tedesco, Giovanni Di Maira, Federico Benetti, Arianna Vanelli

**Affiliations:** 1 Nutrilinea srl, I-21013 Gallarate, Italy; 2 Department of Neurosciences, Sciences of Reproduction and Odontostomatology, University of Naples Federico II, I-80131 Naples, Italy; 3 ECSIN—European Center for the Sustainable Impact of Nanotechnology, EcamRicert SRL, I-35127 Padova, Italy

**Keywords:** bacterial prostatitis, bromelain, pumpkin extract, probiotics, cytokines, inflammation

## Abstract

Among the many factors inducing prostate inflammation, bacterial contribution is potentially underrated according to the scientific community. Bacterial prostatitis is characterized by modifications of the prostatic microenvironment, mainly driven by the immune system. Macrophages play a major role in bacterial prostatitis, secreting a plethora of proinflammatory and chemoattractive cytokines and proteolytic enzymes able to degrade the ECM, so facilitating the invasion of other immune cells. Consequently, macrophages represent a link between bacterial infection and prostate inflammation, as well as being the main target of prostate anti-inflammatory drugs and dietary supplements. This study aims to investigate the effect of a formulation composed of active principles and a probiotic strain with a particular focus on the anti-inflammatory effect in an in vitro bacterial prostatitis model. The results obtained showed that the formulation reduces the inflammatory response of prostatic epithelium induced by bacterial infection. This effect is mediated by the modulation of activated macrophages. Analysis of the cytokines released highlights that the tested formulation is able to reduce the expression of key proinflammatory cytokines involved in the pathogenesis of prostate diseases, in particular prostate cancer, and represents a valuable tool to prevent bacterial prostatitis and ensure favorable prostate health.

## 1. Introduction

Bacterial prostatitis (BP) is an infectious disease that accounts for about 5–10% of all prostatitis cases [1]. Regarding the duration of symptoms, BP is classified as acute and chronic. When symptoms last more than 3 months, BP is classified as chronic bacterial prostatitis. The impact of BP is similar to those patients who have had a myocardial infarction, or who have unstable angina or Crohn’s disease [2]. The pathophysiology of BP remains largely unknown, although some mechanisms of action, such as infectious, immunological, neurological and endocrine have been postulated [3]. BP is commonly caused by Gram-negative bacteria such as uropathogenic *Escherichia coli* (UPEC) even if it has been reported as an emerging prevalence of Gram-positive and atypical bacteria together with anaerobes [4,5]. In particular, UPEC, through the expression of multiple virulence factors, such as fimbriae, lipopolysaccharide (LPS), and toxins, can trigger a series of host inflammatory responses such as cytokine production [6]. The macrophages and neutrophils of the innate immune system provide a first line of defense against these pathogens and are essential for the control of common bacterial infections. Different studies have shown a correlation between inflammatory cytokines and an increased risk for developing prostate cancer [7]. The molecular effects of inflammation on carcinogenesis include alteration of the tumor microenvironment, increased reactive oxygen species level and the upregulation of transcriptional factors [8].

Nutraceuticals are defined as a food or food ingredients that prevent and treat diseases. They contain dietary supplements such as proteins, vitamins and minerals and compounds derived from natural sources [9,10], and it has been demonstrated as contributing to the delay, prevention and treatment of chronic inflammatory diseases [11,12].

In this study, the anti-inflammatory action of an innovative nutraceutical formulation composed of pumpkin extract, bromelain and the probiotic strain *L. rhamnosus* was investigated in an in vitro model of BP. Pumpkin extract has a well-known anti-inflammation action [13], and it is capable of inhibiting testosterone-induced hyperplasia of the prostate. Pumpkin also has a relaxing activity on bladder musculature linked to activation of the nitric oxide (NO) pathway, with reduction in the voiding frequency and an increase in bladder capacity. Bromelain has been employed for the efficient treatment of many inflammation-related disorders, ranging from osteoarthritis and inflammatory bowel diseases to cancer-related inflammation [14]. It exerts analgesic and anti-inflammatory properties due to its ability to influence prostaglandin synthesis. Bromelain also has antibacterial activity linked to the inhibition of bacterial adhesion to the mucous membranes and a synergistic effect with antibiotics. The use of probiotics such as *L. rhamnosus* is a good alternative for the treatment and prevention of urinary tract infection through different mechanisms including attachment to the uroepithelial cells and direct antimicrobial activity [15]. Moreover, *L. rhamnosus* has the ability to effectively inhibit *E. coli* and other Enterobacteriaceae pathogens responsible for bacterial prostatitis. It is also effective in attenuating the inflammation induced at the level of prostatic epithelium and combatting dysbiosis. *Lactobacillus* is an important part of the normal flora, which is commonly found in the mouth cavity, gastrointestinal tract and genitourinary tract. It has been reported that a reduction in the number of *Lactobacillus* increases the risk of urinary tract infections [16]. The formulation and the single active principle were examined in order to investigate the potential anti-inflammatory function in BP, because reducing the inflammatory response in this pathological condition, could help to reduce complications related to prostatitis such as the risk of developing prostate cancer.

## 2. Materials and Methods

### 2.1. Materials

Caco-2 human epithelial colorectal adenocarcinoma cells (ATCC^®^ HTB-37) and human THP-1 monocytes (ATCC^®^ TIB-202™) were purchased from ATCC (Manassas, VA, USA). High glucose Dulbecco’s Modified Eagle Medium (DMEM), Hanks’ Balanced Salt Saline (HBSS), non-essential amino acids (NEAA), L-glutamine, penicillin–streptomycin mix and CellTiter 96^®^ Aqueous One Solution Cell Proliferation Assay (MTS) were purchased from Promega (Madison, WI, USA). Caco-2 human colon adenocarcinoma cell line (ATCC^®^ HTB-37™), LNCaP androgen-sensitive human prostate adenocarcinoma cell line (ATCC^®^ CRL-1740™) and THP-1 (ATCC^®^ TIB-202™) were purchased from ATCC (Manassas, VA, USA). Roswell Park Memorial Institute (RPMI) 1640 Medium, lipopolysaccharide (LPS), diclofenac, were purchased from Sigma-Aldrich (St Louis, MO, USA). Fetal bovine serum (FBS) was purchased from Euroclone (Milan, Italy). Interleukin 1β (IL-1β) ELISA kit was purchased from R and D Systems (Minneapolis, MN, USA). Proinflammatory cytokine-specific array was purchased from Ray Biotech (Peachtree Corners, GA, USA).

### 2.2. Formulation Testing

The anti-inflammatory activity on a bacterial-induced inflammation in vitro model of prostate epithelium was assessed for the active principles described in Table 1, and their mix.

### 2.3. Cell Cultures

#### 2.3.1. Caco-2 Cell Culture

The human epithelial colorectal adenocarcinoma Caco-2 cells (passage 30 to 40) were maintained in Caco-2 cell culture medium (Caco-2 CCM) (DMEM High Glucose medium supplemented with 10% FBS, 2% L-glutamine, 1% NEAA and 1% penicillin–streptomycin mix). The cells were grown in a controlled atmosphere incubator (85% relative humidity, 5% CO_2_ and 37 °C). Caco-2 cells were seeded at 2000 cell/cm^2^ and medium changed every other day. Cells were subcultivated by trypsinization every 7 days when 80–90% confluent.

#### 2.3.2. LNCaP Cell Culture

The human prostate cancer cell line LNCaP cells (passage 25 to 40) were maintained in RPMI-1640 medium supplemented with 10% FBS and 1% penicillin–streptomycin mix. The cells were grown at 37 °C in a humidified atmosphere with 5% CO_2_. Cells were seeded at 10,000 cell/cm^2^ and medium changed every other day. As for Caco-2, cells were subcultivated by trypsinization every 7 days when 80–90% confluent. For vitality and anti-inflammatory experiments, LNCaP cells were seeded in 96-well plates and six-well plates, respectively, at a density of 1 × 10^5^ cell/cm^2^ and allowed to adhere for two days prior to experiments, while for prostate-specific antigen (PSA) experiment, cells were seeded at a density of 50,000 cells/cm^2^ in 24-well plates.

#### 2.3.3. THP-1 Cell Culture

Human THP-1 monocytes passage was maintained in RPMI-1640 medium with glutamate supplemented with 10% FBS, 100 U/mL penicillin and 100 μg/mL streptomycin (GIBCO, Winsford, UK). Cells were cultured at a density of 5 × 10^5^ cells/mL in 5% CO_2_ humidified atmosphere at 37 °C and subcultured twice a week. Macrophage differentiation was induced by incubation with 500 nM phorbol myristate acetate (PMA; Sigma-Aldrich, St. Louis, MO, USA) for 24 h. Culture medium was then replaced and cells cultured for an additional 24 h. For medium conditioning, 6 × 10^6^ cell were seeded in 75 cm^2^ flask, differentiated into macrophages as described before and treated with 1 ng/mL LPS for 6 h. At the end of the LPS treatment, medium was recovered and stored at −80 °C until use.

#### 2.3.4. Bacterial Strain

An amount of lyophilized *L. rhamnosus* was inoculated directly in 5 mL of lactobacilli-selective MRS liquid medium. After 24 h incubation at 37 °C, the obtained suspensions were streaked on MRS agar plate. Following 24 h, a single colony, sampled with a sterile inoculating loop, was inoculated in MRS liquid medium. When *L. rhamnosus* reached its mid-log growth phase (24 h), the concentration of bacteria (Colony Forming Unit (CFU/mL)) was determined by densitometry and confirmed by CFU counts after agar plating of bacterial serial dilutions. *Escherichia coli* and *Staphylococcus aureus* were placed directly on a MacConkey (selective medium for *E. coli*) and mannitol salt agar (MSA) agar plate, respectively, and left to grow for 24 h. As for *L. rhamnosus*, a single colony of each bacterial strain was sampled with a sterile inoculating loop and inoculated in Luria–Bertani (LB) liquid medium. After 24 h (*E. coli* and *S. aureus* mid-log growth phase), the concentration of bacteria (CFU/mL) were determined by densitometry and confirmed by CFU counts after agar plating of bacterial serial dilutions. The growth of both bacteria strains, in liquid (broth) and solid (agar plates) medium, was performed in aerobic condition at 37 °C. Fresh bacteria inocula were prepared before each experiment to ensure treatment consistency.

### 2.4. In Vitro Intestinal Epithelium Model

The intestinal in vitro model used in the present work is based on the human intestinal adenocarcinoma cells Caco-2, cultured as a functional monolayer. Briefly, Caco-2 cells were seeded in multiwell plate in Caco-2 complete medium (CCM) and cultured at 37 °C and 5% CO_2_ in a humidified incubator. The medium was refreshed every other day and cells were allowed to form a monolayer, mature and differentiate up to 21 days.

### 2.5. Medium Conditioning by Probiotic Adhesion to Intestinal Mucosa

Since *L. rhamnosus* is not able to cross the intestinal epithelium in physiological conditions, its anti-inflammatory activity was tested using the conditioned medium following adhesion to intestinal epithelium. *L. rhamnosus* adhesion to the intestinal epithelium in vitro model was performed as described by Candela and colleagues [17]. Before adhesion assay, the intestinal epithelium in vitro model was thoroughly washed with Hank’s Balanced Salt Saline (HBSS) and incubated for 1 h at 37 °C with cell culture medium without antibiotics. Subsequently, 8.6 × 10^8^ CFU/mL (equivalent to a Multiplicity of Infection (MOI) of about 200 bacteria per Caco-2 cell) was added to the apical compartment in cell culture medium without antibiotics and incubated for 3 h in controlled atmosphere incubator (37 °C, 85% relative humidity and 5% CO_2_). Then, Caco-2 monolayers were extensively washed with HBSS to eliminate non-adhered bacteria. Adhered bacteria were left to grow for 24 h and the resulting conditioned medium (i.e., media containing components and metabolic products secreted by *L. rhamnosus*) was sterile-filtered and stored at −80 °C for further application.

### 2.6. In Vitro Model of Innate Immune System

Human THP-1 monocytes (ATCC^®^ TIB-202™) (passage 22 to 32) were maintained in THP-1 cell complete medium (THP-1 CCM) and cultured in controlled atmosphere at 37 °C. Macrophage differentiation was induced by incubating THP-1 with phorbol myristate acetate (PMA) for 24 h. Culture medium was then replaced and cells left to rest for an additional 48 h before exposure to active principles, their mix and the inflammatory stimulus, *E. coli.*

### 2.7. Determination of Cytotoxicity

Cytotoxicity of the active principles and their mix was evaluated on THP-1-based in vitro immune system model. Based on solubility limit, the active principles were resuspended in THP-1 CCM at a concentration of 100 mg/mL for pumpkin extract, 200 mg/mL for bromelain and 125 × 10^6^ CFU/mL for *L. rhamnosus*. The active principles mix was prepared considering the (i) solubility limit of pumpkin extract (100 mg/mL), which is the main component of the formulation, and (ii) the formulation composition information (pumpkin extract 69.76%, *L. rhamnosus* 2.32%, bromelain 27.90%). Macrophage-differentiated THP-1 cells were pretreated for 2 h with increasing concentrations of the active ingredients (0 to 100 mg/mL for pumpkin extract, 0 to 200 GDU/mL for bromelain and 0 to 125 × 10^6^ CFU/mL for *L. rhamnosus*), the mix (0 to 100 mg/mL), the *L. rhamnosus* THP-1 CCM-conditioned medium (0 to 100%) and diclofenac (0 to 400 μg/mL), a well- known anti-inflammatory drug (positive control of anti-inflammatory activity).

After 2 h, the different treatments were refreshed with the addition of the proinflammatory stimulus *E. coli* (1 × 10^6^ CFU/mL), and incubation continued for a further 6 h. After the incubation period, macrophage-differentiated THP-1 cell viability was assessed using LDH cytotoxicity assay to avoid any possible metabolic interferences from bacteria. LDH assay is based on the transformation of a tetrazolium salt into formazan. This transformation is catalyzed by the coupled reaction between the lactate dehydrogenase enzyme (LDH) released by cells due to plasma membrane damage and diaphorase enzyme. The formazan production, proportional to released LDH, can be estimated by measuring its absorbance at 500 nm. The obtained results are expressed as percentage (%) compared to the cell lysis control (positive control; 100% cytotoxicity).

### 2.8. THP-1-Conditioned Medium

THP-1-conditioned media for the investigation of bacterial prostatitis-specific anti-inflammatory activity of the different treatments were obtained by exposing macrophage-differentiated THP-1 to the highest non-toxic concentration of each treatment, according to a two-step protocol. In the first step, macrophage-differentiated THP-1 cells were pretreated for 2 h with the highest non-toxic concentrations of the different treatments, and then (second step) exposed for 4 h to the inflammatory stimulus *E. coli* still in the presence of different treatments. After incubation, THP-1-conditioned media were sterile-filtered and analysed for their interleukin 1β (IL-1β, a well-known proinflammatory cytokine) content with an enzyme-linked immunosorbent assay (ELISA), according to the manufacturer’s instruction.

### 2.9. Evaluation of Anti-Inflammatory Activity

LNCaP cells were exposed for 4 h to conditioned media obtained by exposing macrophage-differentiated THP-1 to the highest non-toxic concentration of the different treatments in presence of *E. coli* (inflammatory stimulus). At the end of exposure, LNCaP cells were washed with DPBS, scraped in ice-cold PBS, centrifugated and lysed by sonication in lysis buffer. Following centrifugation at 10,000× *g* for 15 min, the obtained supernatants were stored at −80 °C. Cytokine release profile was investigated with a proinflammatory cytokine-specific array (Ray Biotech, Peachtree Corners, GA, USA), according to the manufacturer’s instructions. To ensure the cytokine array membrane exposure to the same amount of total proteins, protein content of each LNCaP cell lysate was determined by Bradford assay and LNCaP lysates prepared accordingly (300 μg/mL total proteins). Membranes were incubated in blocking buffer for 30 min and then incubated overnight at 4 °C with rocking with LNCaP cell lysates obtained from the different conditions (non-treated vs. inflamed). Membranes were washed, incubated with biotinylated antibody cocktail diluted in blocking buffer for 2 h at room temperature and rinsed again with wash buffer. HRP-streptavidin was added for 2 h at room temperature, and membranes rinsed and incubated in detection buffer for 2 min at room temperature. Spots related to 23 proinflammatory cytokines were analysed using iBright (Thermofisher, Waltham, MA, USA). The analysis was performed according to the manufacturer’s instructions and results were expressed as fold-change compared to basal inflammation (monolayer exposed to conditioned medium obtained by exposing macrophage-differentiated THP-1 in presence of *E. coli* without active principles or mix).

### 2.10. Statistical Analysis

All data are presented as mean ± standard deviation (SD) of three independent experiments. To determine if statistically significant differences between treatments were present, a *t*-test analysis was performed. The *t*-test is a statistical method used to test differences between two means. The differences between groups were considered significant if *p* < 0.05. For cell cytotoxicity, results are considered significant for cytotoxicity values higher than 30%. All statistical analyses were performed with the OriginLab software (Northampton, MA, USA).

## 3. Results

### 3.1. Impact of Different Bacterial Strains and Bacterial Endotoxin on Innate Immune System In Vitro Model Inflammation

**Figure 1:**
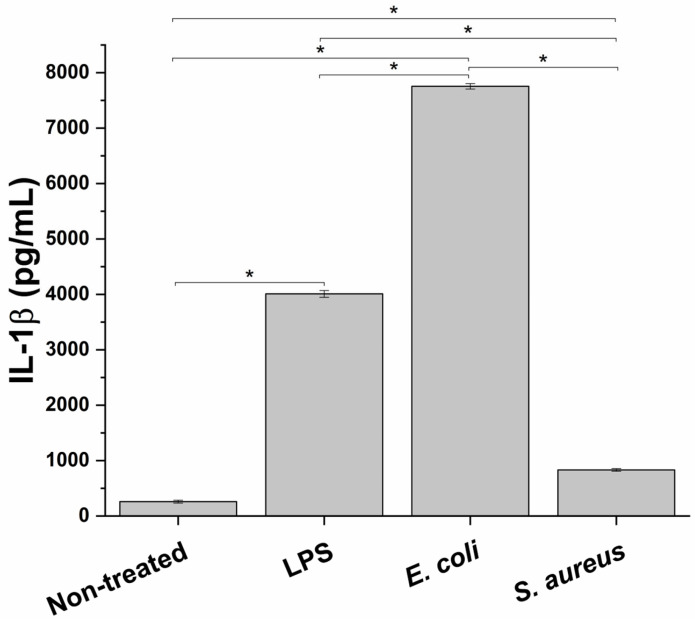
Proinflammatory effect of two bacterial strains, *E. coli* and *S. aureus*, and a bacterial endotoxin (LPS) on the innate immune in vitro system, measured as release of the proinflammatory cy. * *p* < 0.05.

For this study to simulate an in vitro inflammation milieu produced during bacterial infection of the prostate, a macrophage cell line, PMA-differentiated THP-1 cells, representing the innate immune system component, was treated with different infectious stimuli. In particular, in order to test the best experimental conditions to evaluate the anti-inflammatory effect of the formulation, the proinflammatory potential of two different bacterial strains, *E. coli* (Gram-negative bacteria) and *S. aureus* (Gram-positive bacteria), and a bacterial endotoxin LPS, was evaluated. As depicted in Figure 1 at the end of the different incubations, a significant increase in IL-1β release, a typical marker of inflammation, compared to the untreated condition was observed. In particular, *E. coli* triggered the highest proinflammatory response (29.8 ± 0.2 fold-change compared to non-treated cells), followed by LPS and *S. aureus* (15.4 ± 0.2 and 3.2 ± 0.1 fold-change compared to untreated control, respectively). Consequently, *E. coli* was used as a proinflammatory stimulus.

### 3.2. Cytotoxic Effect of Active Principles and Their Mix on In Vitro Cell Model

**Figure 2:**
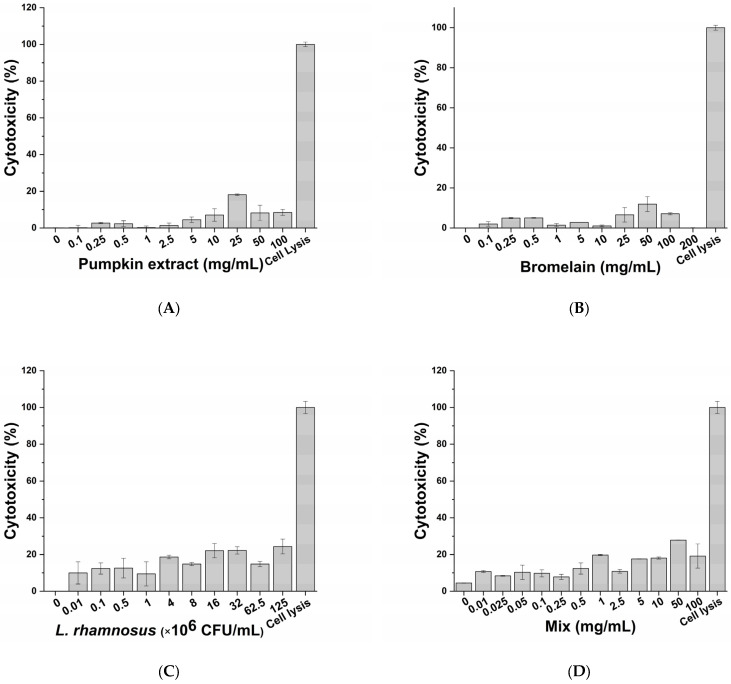

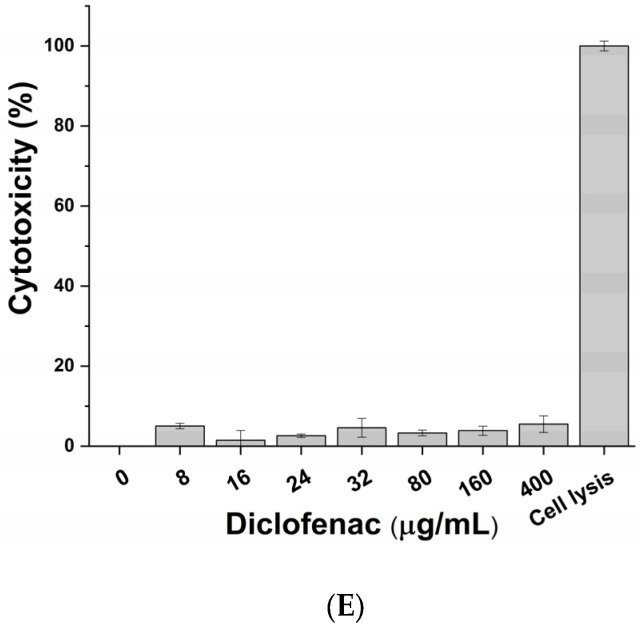
Cytotoxic effect of pumpkin extract (**A**), bromelain (**B**), *L. rhamnosus* (**C**), mix (**D**) and diclofenac (**E**) on the innate immune system in vitro model.

The cytotoxic impact of active principles (pumpkin extract, bromelain and *L. rhamnosus*) and diclofenac (positive control of anti-inflammatory activity) was evaluated on macrophage-differentiated THP-1 according to the protocol described in the Materials and Methods section. No significant adverse effects on macrophages were observed following exposure to the pumpkin extract (Figure 2A), bromelain (Figure 2B), *L. rhamnosus* (Figure 2C), mix (corresponding to the formulation-defined combination of the active principles) (Figure 2D) and diclofenac (Figure 2E) at all tested concentrations. Therefore, considering the results obtained, the inflammatory milieus for exposing prostatic cell lines LNCaP were produced by exposing the macrophage-differentiated THP-1 cells to an inflammatory stimulus and the maximum non-toxic concentration of the single active principles (Table 2).

### 3.3. Anti-Inflammatory Activity of Treatments on Immune System In Vitro Model

**Figure 3:**
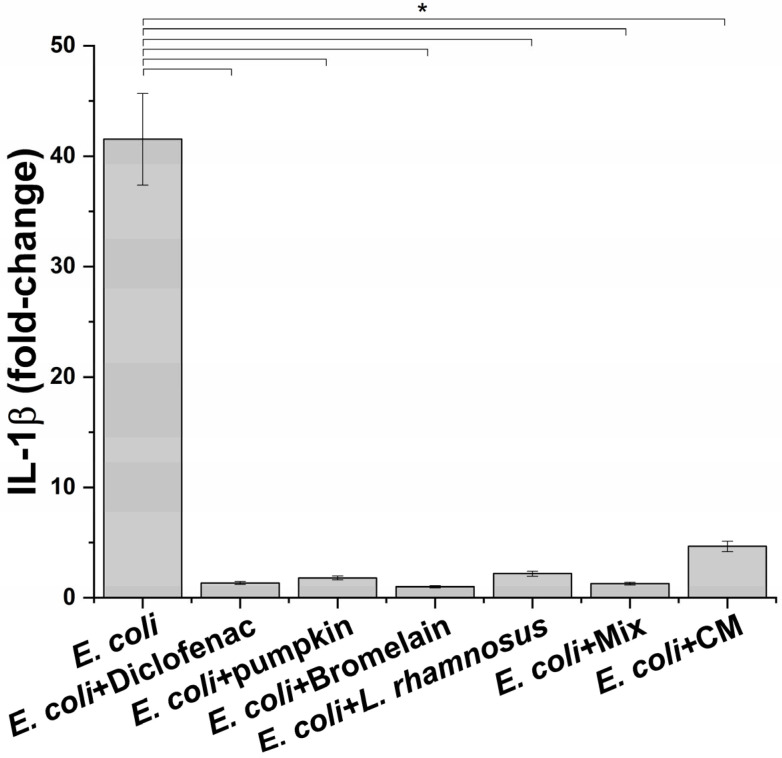
IL-1β release profile after exposure of inflamed macrophages to the indicated treatments. The results are reported as variation (fold-change) compared to the same treatment in absence on *E. coli* (negative control) (mean ± standard deviation). * *p* < 0.05.

Once the best condition to mimic the infection phase was setup, the next step was to evaluate the effect of macrophage-produced inflammatory milieus on prostate epithelium. Before proceeding to this part of the study, the potential anti-inflammatory activity of treatments on macrophages challenged with *E. coli* was determined. For this aim, the level of IL-1β was measured. For the probiotic *L. rhamnosus*, considering that it is highly unlike a direct interaction with prostate epithelium, conditioned medium was also evaluated because it has been reported that the beneficial effect of probiotics in non-intestinal organs occurs through the release of specific molecules [18]. As shown in Figure 3, a significant reduction in the release of the proinflammatory cytokine IL-1β was observed at all tested conditions. For *L. rhamnosus*, treatment with probiotic and conditioned medium induced a comparable reduction in the IL-1β level without affecting cell viability. As expected, the anti-inflammatory drug diclofenac, used as a positive control, caused a reduction in the IL-1β amount in the supernatants of inflamed macrophages.

### 3.4. Impact of THP-1-Conditioned Milieus on Prostate Epithelium In Vitro Model Inflammation

**Figure 4:**
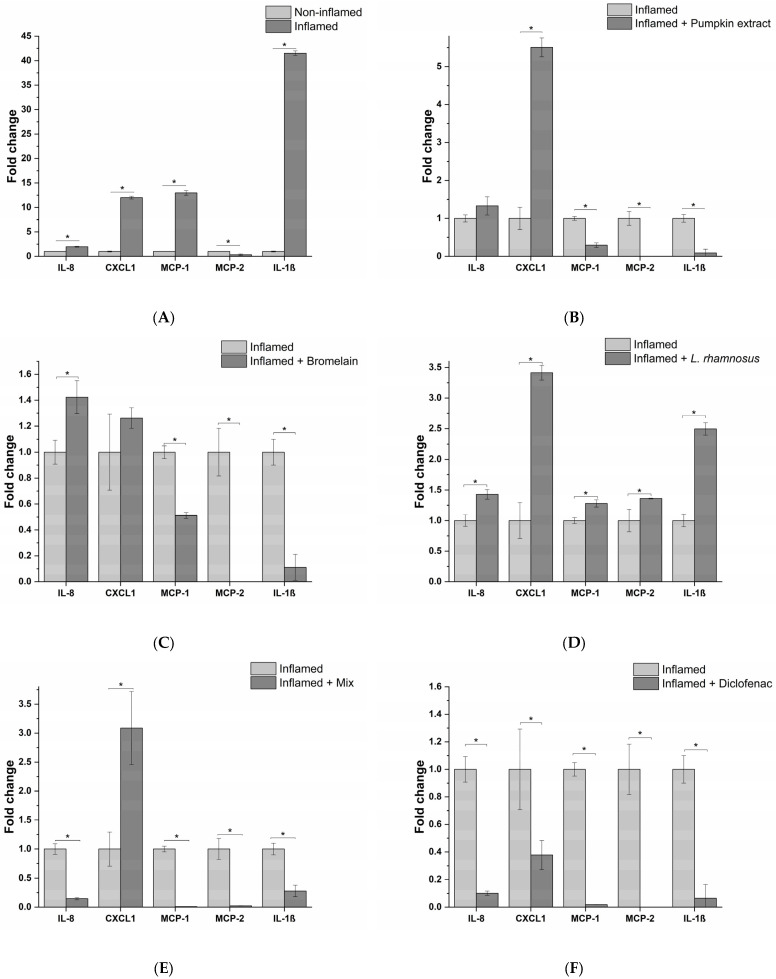
Release profile of IL-8, CXCL1, MCP-1, MCP-2 and IL-1β proinflammatory cytokines following prostate epithelium in vitro model exposure to the different macrophagic milieu: non-treated (**A**), pumpkin extract (**B**), bromelain (**C**), *L. rhamnosus*-conditioned medium (**D**), mix (**E**) and diclofenac (**F**). The results are reported as variation (fold-change) compared to the same treatment with only *E. coli* (Inflamed) (mean ± standard deviation). * *p* < 0.05.

Next, the anti-inflammatory effects of active principles and the formulation were assessed, exposing epithelial prostatic cell line LNCaP with the THP-1-conditioned medium obtained from different treatment conditions. For *L. rhamnosus,* taking into account the results obtained with macrophages, the conditioned medium was tested, instead of the probiotic itself, in order to simulate a physiological condition that could occur at prostatic level. The inflammatory response was investigated by analyzing the release of a panel of 23 proinflammatory cytokines. As evidenced in Figure 4A, cell exposure to the milieu generated by THP-1 macrophages following the exposure to *E. coli*, elicited the release of numerous proinflammatory cytokines, such as (C-X-C motif) ligand 1 (CXCL1), interleukin 8 (IL-8), Monocyte Chemoattractant Proteins (MCP-1 and MCP-2). These cytokines are involved in the activation of proinflammatory responses, in particular, through leukocyte recruitment (i.e., macrophages, monocytes, neutrophils and granulocytes), and subsequent activation and extravasation to the inflammation site, leading to chronic inflammation. Although acting on different proinflammatory targets, the active principles (pumpkin extract, bromelain and *L. rhamnosus*-conditioned medium) showed an effective anti-inflammatory activity since pumpkin extract and bromelain reduced the release of MCP-1, MCP-2 and IL-1β (Figure 4B,C), while the medium conditioned by *L. rhamnosus* reduced the release of IL-8, CXCL1 and MCP-1 (Figure 4D).

Moreover, the release of proinflammatory cytokines was almost completely abrogated by the formulation containing all the active principles, except for CXCL1 (Figure 4E). As confirmation of the obtained results in the in vitro model, the conditioned milieu generated in the presence of *E. coli* and the anti-inflammatory drug diclofenac was tested. As expected, a strong reduction in the release of the proinflammatory cytokines panel analysed was observed (Figure 4F).

## 4. Discussion

Prostatitis is the most common urologic disease in adult males younger than 50 years [19]. Up to 50% of men experience symptoms of prostatitis during their lifetime. Prostatitis has been classified into the following categories: type I, acute bacterial prostatitis; type II, chronic bacterial prostatitis; type III, chronic non-bacterial prostatitis, also known as chronic pelvic pain syndrome (CPPS), which may be “inflammatory” (category III A) or “non-inflammatory” (category III B); and type IV, asymptomatic inflammatory prostatitis [20]. The causes of non-bacterial prostatitis are largely unknown, whereas bacterial prostatitis is caused by infection with uropathogens, especially Gram-negative bacteria, particularly *Escherichia coli*, *Enterobacter*, *Klebsiella*, *Serratia*, *Pseudomonas* and *Proteus* species, although Gram-positive bacteria, particularly *Enterococcus*, can also be responsible for prostatic infection. Pattern recognition receptors (PRRs) are expressed by various innate immunity cells (neutrophils, macrophages, monocytes and dendritic cells) and are able to recognize pathogen-associated molecular patterns (PAMPs), as well as patterns of the external cell walls of bacteria such as the lipopolysaccharides (LPSs) of Gram-negative bacteria and the lipoteichoic acids and peptidoglycans of Gram-positive bacteria [21,22,23]. After the body has recognized microbial pathogens and the innate immune system has been activated, an acute inflammatory response begins with the secretion of various cytokines and chemokines, which stimulate the recruitment of inflammatory cells at the site of prostatic infection [24,25]. Cytokines are key molecules in the immune response involved in the inflammatory cells’ communication. Previous studies of other inflammatory diseases including rheumatoid arthritis, abdominal aortic aneurysms, angiogenesis and psoriasis have shown that cytokines play a central role in inflammatory diseases [26,27].

In this study, the ability to modulate the inflammatory response in bacterial infection of prostatic epithelium by a nutraceutical formulation composed of pumpkin extract, bromelain and the probiotic strain *L. rhamnosus* was evaluated. In order to dissect the molecular mechanisms underlying the inflammatory response in BP, the release of a panel of 23 proinflammatory cytokines was measured following treatment of our in vitro model with the formulation or the single active principles. The results of this analysis showed a wide spectrum of anti-inflammatory activity of the formulation due to the fact that each active principle affects different proinflammatory targets. In particular, pumpkin extract and bromelain reduced the release of MCP-1, MCP-2 and IL-1β. MCP-1 is a key chemotactic agent for monocytes, which differentiate into macrophages once they have reached the inflammation site. MCP-1 could play a role in the pathogenesis of the prostate, in fact it is expressed by prostatic stromal cells in vitro and stimulates the growth of prostatic epithelial cells. The inhibition of MCP-1 or of its receptor (CCR2) blocks the interaction between stromal–epithelial cells, resulting in the inhibition of prostatic cell proliferation. Interleukin-1β (IL-1β) is a proinflammatory cytokine, and has been shown to promote the growth and progression of several solid tumors. Increased IL-1β expression has been found in prostatic inflammation and IL-1β together with IFN-γ, and IL-2 stimulates the secretion of MCP-1 from both prostatic epithelial and stromal cells. Finally, it has been demonstrated that IL-1β is necessary but not sufficient for metastasis of prostate cancer cells [28]. MCP-2 is a CC chemokine with high homology to MCP-1. MCP-2 is able to recognize several receptors including CCR1 [29], CCR2 [30,31], CCR3 [32] and CCR5 [33]. As a consequence, MCP-2 represents a pluripotent chemokine, and in the case of bacterial infections, its expression is increased, favouring the recruitment and activation of monocytes [34], lymphocytes [35], NK cells [36] and basophils [33] in the site of infection. On the other hand, the medium conditioned by *L. rhamnosus*, as well as reducing the release of MCP-1, induces a decrease in IL-8 and CXCL1 levels.

IL-8 exerts chemotactic effects on white blood cells [37,38]. Its expression is regulated by inflammatory mediators such as IL-1β and environmental stress, and steroid hormones [9,39]. IL-8 stimulates proliferation, invasion, cell survival and chemoresistance, elevated IL-8 serum levels have been found in patients with prostate cancer, and it has been associated with poorer outcomes [40,41]. CXCL1 belongs to the CXC family chemokine and it has been found to be overexpressed in prostate cancer. CXCL1 acts as a paracrine factor promoting prostate cancer metastasis through cross-talk among multiple cell types within the tumor microenvironment [42]. Moreover, a recent study demonstrated that obese patients with prostate cancer have an increased expression of CXCL1 and its overexpression is linked to obesity-dependent tumor adipose stromal cells’ recruitment and, ultimately, promotes the progression of prostate cancer [43].

## 5. Conclusions

Altogether, the results obtained highlight the potential benefit of the formulation tested on bacterial prostatitis treatment; in particular, the active principles present in the formulation target key cytokines released by pathogen-activated macrophages and reduce the subsequent prostate cell inflammatory response. This finding is noteworthy because cytokines produced by innate immune cells represent an attractive target for therapeutic intervention against several inflammatory malignancies; therefore, the tested formulation could represent a useful therapeutic tool to tackle bacterial prostatitis that is often associated with prostate cancer onset and progression.

## Figures and Tables

**Table 1:** Description of analysed active ingredients. GDU: Gelatin Dissolving Units.

Active Principles	Form
Pumpkin d.e. (tit. 40% in fatty acids)	Powder
Bromelain (2400 GDU/g)	Powder
*L. rhamnosus* (LRH020 300 B/g)	Powder

**Table 2:** Treatment concentration used for the production of inflammatory milieus used for treating the prostate epithelium in vitro model.

Treatment	Highest Non-Cytotoxic Concentration
Pumpkin extract	100 mg/mL
Bromelain	200 mg/mL
*L. rhamnosus*	125 × 10^6^ CFU/mL
Mix	100 mg/mL
Diclofenac	400 μg/mL

## Data Availability

Not applicable.
